# A Systematic Review of the Epidemiology, Immunopathogenesis, Diagnosis, and Treatment of Pleural TB in HIV- Infected Patients

**DOI:** 10.1155/2012/842045

**Published:** 2012-03-14

**Authors:** A. Aljohaney, K. Amjadi, G. G. Alvarez

**Affiliations:** ^1^Department of Internal Medicine, Faculty of Medicine, King Abdulaziz University, P.O. Box 80215, Jeddah 21589, Saudi Arabia; ^2^Divisions of Respirology and Infectious Diseases, Ottawa Hospital Research Institute, at the Ottawa Hospital, University of Ottawa, 725 Parkdale Avenue, Ottawa, ON, Canada K1Y 4E9

## Abstract

*Background*. High HIV burden countries have experienced a high burden of pleural TB in HIV-infected patients. *Objective*. To review the epidemiology, immunopathogenesis, diagnosis, and treatment of pleural TB in HIV-infected patients. *Methods*. A literature search from 1950 to June 2011 in MEDLINE was conducted. *Results*. Two-hundred and ninety-nine studies were identified, of which 30 met the inclusion criteria. The immunopathogenesis as denoted by cells and cytokine profiles is distinctly different between HIV and HIV-uninfected pleural TB disease. Adenosine deaminase and interferon gamma are good markers of pleural TB disease even in HIV-infected patients. HIV-uninfected TB suspects with pleural effusions commonly have a low yield of TB organisms however the evidence suggests that in dually infected patients smear and cultures have a higher yield. The Gene Xpert MTB/RIF assay has significant potential to improve the diagnosis of pleural TB in HIV-positive patients. *Conclusions*. Pleural TB in HIV-infected patients has a different immunopathogenesis than HIV-uninfected pleural TB and these findings in part support the differences noted in this systematic review. Research should focus on developing an interferon gamma-based point of care diagnostic test and expansion of the role of Gene Xpert in the diagnosis of pleural TB.

## 1. Background

The World Health Organization reported 1.1 million new cases of TB among HIV-infected persons in 2009 [1]. High HIV prevalence regions have experienced a greater burden of extrapulmonary TB [1]. Extrapulmonary TB is more common in HIV-infected patients compared with patients without HIV infection [2, 3] and its incidence has doubled since the beginning of the HIV pandemic [4]. Furthermore, pleural TB is the second most common form of extrapulmonary TB after peripheral lymph nodes in HIV-infected patients [4]. Pleural TB in the context of HIV continues to provide formidable challenges to clinicians around the world. In this systematic review, we report the epidemiology, immunopathogenesis, clinical presentation, diagnosis, and management of tuberculous pleural effusions in HIV-positive patients and explore some of the important differences between them and HIV-uninfected patients as well as how these findings could be applied in resource poor settings.

## 2. Methods

The PRISMA guidelines for systematic reviews [34] were used to formulate this paper.

### 2.1. Eligibility Criteria

Studies were included if all of the following criteria were met: (1) all study types except case reports published in English, French, Spanish, and Arabic and (2) pleural tuberculosis were defined by one of the following features: (a) positive culture of the pleural fluid or pleural biopsy specimen or both for *M. tuberculosis,* (b) positive sputum culture for *M. tuberculosis* and pleural effusion, positive acid fast bacilli (AFB) stain of the pleural fluid, or pleural biopsy specimen, or (c) granuloma on pleural biopsy with no alternative diagnosis and improvement on antituberculous therapy. (3) HIV infection documented by at least serological testing.

### 2.2. Search Strategy

A literature search in MEDLINE was conducted by one of the investigators (AA). Articles were limited to English, French, Spanish, and Arabic published between 1950 to June 2011. Using Boolean operator “and”, we combined the following research themes (1) (“Tuberculosis, Pleural/complications" [Mesh] or “Tuberculosis Pleural/diagnosis" [Mesh] or “Tuberculosis, Pleural/drug therapy" [Mesh] or “Tuberculosis, Pleural/enzymology" [Mesh] or “Tuberculosis, Pleural/epidemiology" [Mesh] or “Tuberculosis, Pleural/microbiology" [Mesh] or “Tuberculosis, Pleural/mortality" [Mesh] or “Tuberculosis, Pleural/pathology" [Mesh] or “Tuberculosis, Pleural/radiography" [Mesh] or “Tuberculosis, Pleural/surgery" [Mesh] or “Tuberculosis, Pleural/therapy" [Mesh]) or pleural tuberculosis [Text Word] and (2) “HIV Infections" [Mesh] or HIV infection [Text Word].

We also scanned the bibliographies of key articles to identify additional studies. The literature search was crosschecked by the university librarian to ensure reproducibility and that no other citations existed.

### 2.3. Study Selection and Data Collection Process

One reviewer (AA) screened the titles and abstracts of identified records. Articles were retrieved for full text review if they contained any information related to pleural tuberculosis and HIV infection. Full text review of these articles was performed by two reviewers (AA, GGA) independently using predefined case report forms. The two reviewers then met to discuss each article using the predefined case report forms. Differences were resolved by consensus. Variables that were collected included: incidence, prevalence, immunopathogenesis, diagnosis, management, and treatment of pleural TB in HIV-infected patients.

## 3. Results

### 3.1. Study Selection

**Figure 1:**
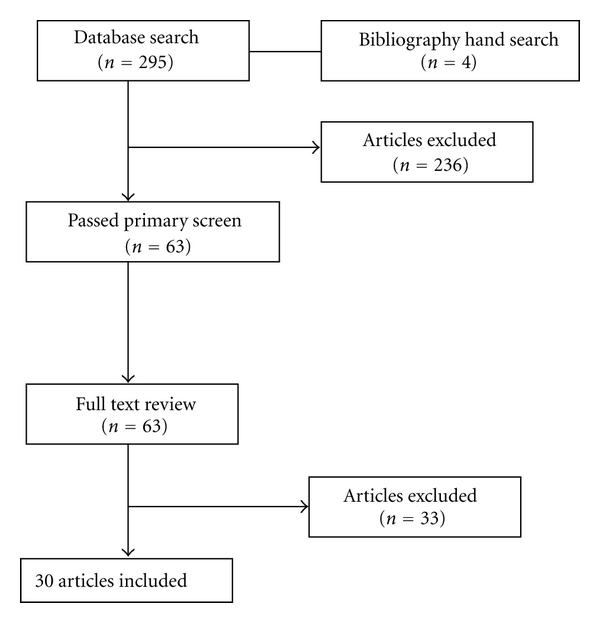
Identification, screening, eligibility, and inclusion of studies.

Figure 1 outlines the identification, screening, eligibility, and inclusion of studies in this systematic review. The initial search strategy identified 295 potentially relevant articles and four additional studies from review of bibliographies. 63 articles were chosen based on titles and abstracts. 30 articles fulfilled the eligibility criteria and 33 articles were excluded after full text reviews. Selection and information bias, lack of uniform reporting, and inclusion of low methodological quality studies prevented a formal meta-analysis.  Table 1 shows the characteristics of the 30 included studies.

## 4. Epidemiology of Pleural TB in HIV-Infected Patients

The prevalence rate of HIV-pleural tuberculosis was highest in the African regions compared to other regions (Table 2). The highest rate was in Zimbabwe where 85% of patients with tuberculous pleural effusions in one study were HIV-positive [16] and the lowest rate was seen in another study done in Spain at 10% [15]. Although the study populations were heterogeneous, it can be extrapolated that due to the fact that the WHO estimates that 37% of dually infected incident cases came from the African region [1], it would be expected that more pleural TB would be seen in these regions. Males were more frequently infected with HIV and there was no gender differences noted between HIV-positive compared with HIV-uninfected individuals [18, 23, 24].

## 5. Immunopathogenesis of Pleural TB in HIVInfected Patients

Pleural TB has been characterized as a systemic inflammatory response which in some HIV-uninfected patients has been documented to resolve without the use of antibiotics [36]. This systemic inflammatory response likely contributes to the constellation of symptoms seen in these patients which can be more severe than in pulmonary TB [37]. The development of a pleural effusion in immunocompetent hosts is associated with an intense cell-mediated immune response with infiltration of CD4 T cells and production of high levels of proinflammatory cytokines such as gamma interferon (IFN-) and tumor necrosis factor alpha (TNF-*α*) [36, 38]. In contrast, the immunopathogenesis of pleural TB in HIV-infected patients is different because of the CD4 T cell depletion and subsequent reduction in antigen-specific cytokine responses [39]. HIV-infected patients have decreased numbers s of CD4+ T cells and cytokine responses to TB [40–43]. When these immunological components are lacking the key protective immune response is significantly weakened. T helper 1 type cells secrete TNF*α*, IFN*γ*, IL-2, and IL12. These cells are important in the delayed type hypersensitivity reaction and in activating macrophages in pleural TB. Furthermore, granuloma formation is mediated by CD4+ T cells of the T helper 1 type.

### 5.1. Histopathology

In an early study done in Tanzania [35], 36 HIV TB pleural biopsies were compared with 21 HIV-uninfected TB-pleural biopsies. Histological characterization of the tissue reaction in pleural biopsies were examined as follows: reactive (well-formed granulomas with caseous necrosis, epitheliod cells, giant cells, scarce acid fast bacilli, or undetected AFB), hyporeactive (poorly formed granulomas with noncaseous necrosis, few epithelioid cells or macrophages, and no giant cells, AFB were easily seen) and non-reactive (no true granuloma formation, noncaseous necrosis with nuclear debris and neutrophils, no giant cells and numerous AFB). Although CD4 counts were not done in this study, HIV-positive patients had significantly more pleural biopsies demonstrating the hyporeactive and nonreactive patterns than HIV-uninfected patients (14/36 versus 2/21 *P* < 0.02) and their level of immunodeficiency was worse than those with reactive patterns among the HIV-infected patients as evidenced by a greater number of AIDS related complications. Furthermore, hyporeactivity in the HIV group seemed to show a trend towards a mortality risk (3/11 versus 1/18 deaths). In contrast, a case series of 12 patients (6 HIV-positive and 6 HIV uninfected) more necrotic granulomas were seen in HIV-positive patients with pleural TB [24].

In a case series of three HIV-pleural TB cases [28], a significant number of mesothelial cells were noted in the pleural fluid. Commonly, few mesothelial cells are seen in the pleural space in patients with pleural TB since it is believed that there is extensive chronic inflammation that covers the mesothelium preventing it from exfoliating these cells into the pleural fluid.

### 5.2. Cytokine Profiles in Pleural TB in HIV-Infected Patients

Cytokine profiles in the pleural fluid of pleural TB in HIV-infected patients versus TB-pleural patients showed a mostly Th1 (or proinflammatory, instead of the Th2 or anti-inflammatory) cytokine profile [31]. No differences were found in the levels or patterns of cytokines in pleural fluid between HIV-positive and negative patients except for higher IL-8 levels seen in dually infected individuals [31]. In another study, comparing HIV-uninfected and HIV-positive pleural TB patients, 29 cytokines were measured in the plasma and pleural fluid. IL-1*β*, IL-10, and TNF*α* were significantly decreased in the pleural fluid of HIV-positive pleural TB patients [32]. In addition, two proinflammatory markers, CXCL10/IP-10 and CCL3/MIP-1*α*, measured in the plasma were characteristic of pleural TB [32]. However, HIV infection affected the diagnostic accuracy as evidenced by a shift in cutoff values used, resulting in increased specificity at the expense of decreased sensitivity in pleural TB in HIV-infected persons compared to pleural TB in HIV-noninfected persons [32]. Other studies have shown that the levels of TNF*α* and MCP-1 were significantly elevated in the pleural fluid compared with autologous plasma in dually infected individuals [33]. MCP is a chemokine produced by fibroblasts and mesothelial cells and is a chemotactic agent for monocytes and lymphocytes. Both TB and HIV products can induce the production of MCP-1. Furthermore, transcription activation of HIV-1 in situ can be significantly reduced by neutralization of MCP-1; however, only when TNF*α*. was not neutralized suggesting a possible relationship between MCP-1/TNF*α* [33].

Necrotic granulomas from pleural biopsies done in coinfected patients with pleural disease showed significantly elevated TNF *α*-positive cells [24]. Although the authors acknowledged that this may not have equated to increased levels of the cytokine, the presence of this marker on the cells is an important difference which may explain the progression of pleural TB in HIV-infected patients in HIV-infected patients since apoptotic activity has been linked with TNF*α* and its continued presence is detrimental to the cellular environment [44] resulting in caseating necrosis. In another study, TNF*α* was elevated in HIV-pleural TB compared to HIV-uninfected pleural TB but the difference was not statistically significant [14]. In this same study, IFN*γ* was significantly elevated in serum and pleural fluid in HIV-pleural TB compared to HIV-uninfected pleural TB and it was suggested that CD8+ T cells could be the source which is supported by another study showing a relative increase in CD8+ T cells in HIV-positive patients with pleural TB [14]. However, mycobacterial replication was not controlled in the pleural space despite high levels of IFN*γ*. No difference was seen in the IL-10 levels in the pleural space between the two groups [14]. Another study [27] suggested apoptosis and levels of IFN*γ* are increased in HIV-infected patients with pleural TB; however, this finding was not unique to HIV TB and seen in pleural TB alone.

## 6. Clinical Features of Pleural TB in HIV-Infected Patients

HIV-infected patients with pleural tuberculosis were more likely to present with fever, [13, 15, 17], dyspnea [17], cough [15] and significant weight loss [15] in comparison with HIV-uninfected patients. Furthermore, systemic symptoms and signs such as fatigue, night sweats, diarrhea, lymphadenopathy, splenomegaly, and hepatomegaly were more common in HIV-infected patients [13]compared to HIV-uninfected patients [45]. Bilateral pleural effusions were also more frequently reported in HIV-positive patients [5] but the size or location of pleural effusions were comparable [5, 13, 15]. HIV-positive patients that present with pleural TB are generally sicker than non HIV-uninfected individuals as reflected by the increased frequency of systemic symptoms. Symptoms alone are limited in their ability to diagnose pleural TB in HIV-positive patients due to their non specific nature [13]. The severity of symptoms at presentation may reflect the higher degree of impairment in the immune system in HIV-positive patients which leads to more disseminated forms of the disease resulting in more advanced disease at presentation.

## 7. Diagnosis

### 7.1. Pleural Fluid

Pleural fluid examination showed increased mesothelial cells [28], decreased albumin and higher gamma globulinlevels in HIV-infected patients [17]. Lymphocyte predominant effusions [15], LDH, protein and glucose levels did not differ between dually infected patients with pleural TB [16].

### 7.2. Mycobacterium TB Identification in Pleural Fluid

Ziehl-Neelsen (ZN) stain [13, 15], liquid culture using BACTEC [14, 15] and Löwenstein-Jensen (LJ) cultures [11, 13, 15, 18, 26, 27, 41] consistently provided a higher yield in HIV-infected individuals compared to HIV-uninfected individuals. In one study [13], the more immunocompromised the patient, the higher chance of finding TB organisms in the pleural fluid and the pleura itself. A CD4 count of <200 × 10^6^/L was associated with a positive pleural fluid smear (37% versus 0% *P* = 0.0006) and biopsy Ziehl-Neelsen stain (35% versus 7% *P* = 0.021)[13]. The TB yield of different tests used in tuberculouspleuritis in HIV-infected patientsis shown (Table 3).

Although many studies looking at nucleic acid amplification tests (NAAT) applied to pleural TB in HIV-infected patients have been done [45] they have shown considerable variability. However, the new gene xpert MTB/RIF assay is a significant advance in point of care molecular diagnostic biology [46] that could provide a significant improvement in diagnosing pleural TB in HIV-infected patients with the added benefit that rifampin resistance is also detected by the assay and the result can be obtained in 2 hours. Although no studies have been published in HIV-infected patients with pleural TB, a retrospective analysis of specimens sent to a national reference laboratory in Germany for mycobacteria [47] studied various specimen types including 113 pleural fluid samples for which the specificity was calculated at 98.1% and the sensitivity was not calculable.

### 7.3. HIV Identification in Pleural Fluid

HIV viral load in pleural fluid in dually infected patients has been demonstrated to be higher in the pleural fluid when compared to plasma [31]. Another study [33] showed that transcriptional activity of HIV-1 was significantly higher in pleural fluid mononuclear cells (PFMC) compared to peripheral blood mononuclear cells (PBMC). Increased HIV viral production was seen in the pleural space of HIV-pleural TB patients from activated HLA-DR mononuclear cells including lymphocytes and CD14+ macrophages [41]. Another study [25] suggested that HIV-positive patients with pleural TB showed higher HIV viral replication and heterogeneity which then migrated to the blood increasing systemic HIV heterogeneity. The excess viral loads seen in the pleural fluid of HIV-TB-pleural patients make it an important site to gain a better understanding of whether the increased levels of virus seen in the pleural fluid of dually infected patients may affect HIV progression in these patients. The development of targeted inhibitors of viral replication in dually infected patients could offer new insight.

### 7.4. Pleural Biopsy

Ziehl-Neelsen stain of pleural biopsy carries significantly higher yield in HIV-infected patients than non HIV in two studies [13, 26] and not significantly higher in other studies [17, 35]. The utility of histological examination of pleural biopsy was high in HIV-infected patients where granulomatous inflammation or caseous necrosis was detected in 52%–92%. This finding was comparable with HIV-uninfected patients [11, 13, 15, 17, 26].

### 7.5. Sputum Culture

Sputum culture yield in pleural tuberculosis was reported to be higher in HIV-infected patients, however this was not statistically significant [13, 26]. One of these studies [26], demonstrated that the yield of sputum cultures using sputum induction in dually infected patients with pleural TB who could not produce sputum spontaneously was high even in patients with no pulmonary findings on chest radiographs.

The use of the pleural fluid or biopsy smear to diagnose pleural TB in HIV-infected patientsremains an effective tool which is widely available and inexpensive especially in regions with limited resources. Clinicians often do not bother sending pleural fluid for smear and culture due to their low yield in the HIV-uninfected patient population; however, the evidence suggests that in dually infected patients smear and cultures should play an important role in the diagnostic process (Table 3). The pleural fluid and pleural biopsy TB culture yield is higher in HIV-infected patients. This may suggest that a higher bacillary burden is seen in the pleural space in HIV-infected patients because TB might cross from the lung parenchyma to the pleural space with greater ease as a result of an impaired immune response in the pleural space. Furthermore, the use of bedside inoculation of pleural fluid in BACTEC liquid medium provides a better sensitivity and faster results in HIV coinfection [15]. All of these classic forms of diagnosing pleural TB are limited in the prolonged time it takes to obtain results and also from the limited laboratory access in the clinic/hospital where the patients are assessed. These diagnostic delays result in significant morbidity and mortality of patients. In regions with limited resources, a point of care test which could be done at the bedside on pleural fluid would have a significant impact on the management and outcome of these patients.

### 7.6. Adenosine Deaminase (ADA)

Adenosine deaminase (ADA) a T lymphocyte enzyme that catalyzes the conversion of adenosine and deoxyadenosine to inosine and deoxyinosine, respectively. Two different molecular forms of ADA, ADA 1, and ADA2 have been identified [48]. ADA1 is found in all cells, with its greatest activity in lymphocytes and monocytes. ADA2 isoenzyme is found mainly in monocytes/macrophages. Most of the ADA found in tuberculous pleural fluid is ADA2, whereas most of the ADA found in other pleural fluids is ADA1. Testing ADA levels in the pleural fluid is an easy, inexpensive, and useful test to establish the diagnosis of pleural TB. ADA retains its high utility in all HIV-infected patients [16] even patients with low CD4 counts [22]. ADA improves the accuracy of diagnosis in HIV-infected patients with pleural TB [16, 17, 22]. In all HIV-infected patients regardless of CD4 counts, the sensitivity of ADA was 94% when the cutoff value of 30 u/l was used and specificity of 95% [22]. The positive likelihood ratio was 18.9 and the negative likelihood ratio was 0.06 [22]. The sensitivity wasalso highat 96% when the cutoff value used was 60 u/l in HIV-infected patients [16]. These results were comparable to HIV-uninfected patients [16].

However, it should be noted that the sensitivity and specificity of ADA vary according to the different cutoff levels and also to different TB population prevalence. ADA measurement has a limited value in regions of low TB prevalence [49] as it can also be elevated in patients with empyema, lymphoma, lung cancer, rheumatoid arthritis, systemic lupus erythematosus, brucellosis, and Q fever [50]. In a high HIV and TB prevalence region, the use of ADA at a higher cut point (47 IU/L) compared to the standard cut point (30 IU/L) in which a subgroup of HIV tested patients were studied, it was noted that ADA increases its specificity while use of the standard cut point (30 IU/L) results in loss of specificity but an increase in its sensitivity and thus improves its ability to rule out disease.

A possible explanation for the high levels of ADA even in HIV-infected patients with low CD4 counts may be related to the fact that monocytes are not significantly affected by HIV coinfection and they are the primary cells responsible for the production of isoenzyme ADA-2. Riantawan et al. [16] documented the best cutoff of ADA at 60 U/L in HIV-infected patients which provides sensitivity of 95% and specificity of 96%. Liang QL et al. [51] published a meta-analysis in mostly HIV-uninfected patients which included 63 studies documenting sensitivity and specificity of pleural ADA in the diagnosisof pleural TB to be 92 and 90%, respectively [51]. The positive likelihood ratio was 9.03, the negative likelihood ratio was 0.10, and the diagnostic odds ratio was 110.08 [51].The most widely accepted cutoff value for pleural fluid ADAis 40 U/l [50]; however, this cutoff will likely need to be higher in the HIV population.

### 7.7. Interferon Gamma

Interferon gamma levels in pleural tuberculosis were significantly higher in both serum and pleural fluid of HIV-positive patients when compared with HIV-uninfected patients [14]. There was no statistically significant correlation between blood CD4 cell count and the level of pleural INF-gamma [14, 21] but a positive correlation with pleural fluid viral loads was noted (correlation coefficient 0.54, *P* = 0.02) [14]. Another study documented interferon gamma sensitivity of 99% and specificity of 98% using 3.7 U/mL cutoff point which did not differ between HIV-positive and HIV-uninfected patients [20]. In a meta analysis of 27 studies evaluating the role of interferon gamma release assays (T-SPOT.TB1, QFT-G-IT), and Tuberculin Skin Test (TST) in diagnosing active tuberculosis [52], 5 studies used IGRA tests for the diagnosis of pleural TB [8, 21, 53–55]. None of these studies tested all of the patients entering the respective studies for HIV and all were limited by small sample sizes. However, one study [8] did test most of the cohort for HIV (51/67) and managed to confirm that even in a high HIV burden region, unstimulated pleural fluid interferon gamma levels measured in TB suspects were found to be highly sensitive and specific for distinguishing pleural TB from non-TB effusions [8]. In the same study it was also shown that because many HIV-positive patients likely have paucicellular pleural inflammation, adequate volumes of pleural fluid need to be obtained (>20 mL) in order to obtain adequate number of cells to analyze [8]. Interferon gamma inducible protein of 10 kDa (IP-10) and lipoarabinomannan (LAM) mycobacterial antigen-detection assay were not useful in discriminating pleural TB patients versus non-TB patients in a subset of HIV-infected patients; however, future work should focus on validating IP-10 ability to rule out pleural TB [9]. The impact of immunosupression seen in HIV infection on the diagnostic accuracy of pleural mononuclear cells and their capacity to secrete interferon gamma requires further study in larger studies to confirm the accuracy of the findings presented.

Pleural TB in HIV-infected patients remains a challenge to diagnose because often sputum smears and cultures are negative. Thoracentesis is commonly performed to examine the composition of the pleural fluid. However, biochemical, histological and microbiological examination is usually limited in low-resource countries. A single point of care test that could be applied to pleural fluid would have enormous impact on the diagnosis and management of these cases.

## 8. Treatment

There is no evidence to suggest that HIV-pleural tuberculosis should be treated differently than pulmonary TB. Two months of isoniazid, rifampin, pyrazinamide and ethambutol followed by 4 months of isoniazid and rifampin for susceptible organisms to all first line drugs are the international standard [56].

It is recommended that antiretroviral therapy should be delayed for 2 months unless the CD4 < 100× 10^6^/L to prevent immune reconstitution syndrome which could occur in one-third of patients [57]. The use of rifabutin instead of rifampin is recommended to avoid drug interaction with protease inhibitors and most nonnucleoside reverse transcriptase inhibitors, with the exception of efavirenz. Even so, rifampin-containing regimens can be prescribed if the selected antiretroviral drugs include efavirenz and two nucleoside transcriptase inhibitors (e.g., tenofovir and emtricitabine) [50]. In one randomized controlled study of 197 patients with HIV associated pleural TB, the administration of prednisolone as adjunctive therapy to standard first line TB therapy did not result in a survival benefit and although it was associated with faster resolution of the TB it was also associated with an increased risk of Kaposi sarcoma [29]. The patients in this study were significantly immunocompromised and the effects of steroids on HIV-infected patients with CD4 counts >200 × 10^6^/L still need to be assessed. A recent Cochrane review found no clear evidence to support the use of adjunctive corticosteroids in all persons with TB-pleural effusion [58].

## 9. Quality of Included Studies

Many of the studies had small sample sizes and did not have consecutive recruitment of patients which may have resulted in selection and information bias. Furthermore, the study design limited the quality of several studies with only one randomized controlled trial, several prospective cohorts, some retrospective chart reviews and several case series. Another significant limitation is the fact that many of the HIV-infected patients had different levels of immunosuppression (different levels of CD4 cells) which could affect the immunopathogenesis studies.

## 10. Reasons for Exclusion of Studies

Many of the studies that were excluded from this analysis did not establish the diagnosis of pleural TB or HIV accurately. One common reason for exclusion was that the patients in the HIV-uninfected group were not tested for HIV and assumed to be HIV uninfected. Some of the earlier studies opted to make a clinical diagnosis of HIV instead of getting serological testing which may have not been available at the time of the study. Other studies categorized pulmonary TB in the same category as pleural TB, whereas we categorized it as extrapulmonary disease with specific criteria depicted in the inclusion criteria section. Several studies were excluded because they were case reports.

## 11. Future Research

Pleural TB in HIV-infected patients has a different immunopathogenesis than HIV-uninfected pleural TB and these findings in part support the diagnostic differences seen in the yield of TB in the pleura in HIV-infected patients compared to HIV-uninfected patients. More research is needed in the field of immunopathogenesis of HIV-pleural TB disease as it offers an important microcosm where the bacteria and virus interact allowing the study of this complex interaction between HIV and TB to be further elucidated. Furthermore, the immunopathogenic differences may help develop a better interferon gamma-based point of care test that could be used at the bedside on pleural fluid in low-resource countries with high HIV prevalence. The Gene Xpert MTB/RIF assay technology applied to pleural fluid is a formidable area of research which could have enormous impact on the early diagnosis of pleural TB in HIV-positive patients and should be deemed a research priority in this field.

## Figures and Tables

**Table 1:** Characteristics of included studies.

Study/reference	Year	No of pleural TB patients		Design	Recruitment of patients
		HIV+	HIV−		
Aderaye et al. [5]	1996	8	54	Prospective cohort	Consecutive
Batungwanayo et al. [6]	1993	28	82	Prospective cohort	Consecutive
Cordero et al. [7]	1995	12	107	Retrospective chart review	N/A
Dheda et al. [8]	2009	38	13	Prospective cohort	Consecutive
Dheda et al. [9]	2009	20	31	Prospective cohort	Consecutive
Domoua et al. [10]	2007	30	17	Prospective cohort	Consecutive
Elliott et al. [11]	1993	57	13	Prospective cohort	Consecutive
Frye et al. [2]	1997	22	169	Retrospective chart review	N/A
Gil et al. [12]	1995	10	93	Retrospective chart review	N/A
Heyderman et al. [13]	1998	63	11	Prospective cohort	Consecutive
Hodsdon et al. [14]	2001	66	29	Prospective cohort	Consecutive
Luzze et al. [15]	2001	109	33	Prospective cohort	Consecutive
Riantawan et al. [16]	1999	37	52	Prospective cohort	Consecutive
Richter et al. [17]	1994	65	47	Prospective cohort	Consecutive
Richter et al. [18]	1994	49	26	Prospective cohort	Consecutive
Trajman et al. [19]	1997	13	30	Cross-sectional retrospective	N/A
Villena et al. [20]	1996	9	41	Prospective cohort	N/A
Baba et al. [21]	2008	16	2	Retrospective case series	N/A
Baba et al. [22]	2008	145	20	Retrospective case control	N/A
Baba et al. [23]	2008	5	23	Prospective cohort	Consecutive
Bezuidenhout et al. [24]	2009	6	6	Case series	N/A
Collins et al. [25]	2007	8	0	Case series	N/A
Conde et al. [26]	2003	13	71	Prospective cohort	Consecutive
Hirsch et al. [27]	2001	16	8	Prospective cohort	Consecutive
Jones et al. [28]	2000	3	0	Case series	N/A
Elliott et al. [29]	2004	197	0	Randomized controlled Trial	Consecutive
Tshibwabwa-Tumba et al. [30]	1997	159	68	Prospective cohort	Consecutive
Toossi et al. [31]	2011	20	20	Prospective cohort	Consecutive
Siawaya et al. [32]	2009	12	11	Case series	N/A
Mayanja-Kizza et al. [33]	2009	20	0	Case series	N/A

N/A: not mentioned in the methods section of the paper.

**Table 2:** Reported prevalence rates of HIV-pleural tuberculosis across various studies/countries.

Study/year	Country	Prevalence
Heyderman et al. 1998 [13]	Zimbabwe	85%
Batungwanayo et al. 1993 [6]	Rawanda	80%
Luzze et al. 2001 [15]	Uganda	80%
Domoua et al. 2007 [10]	Ivory Coast	63%
Richter et al. 1994 [18]	Tanzania	58%
Riantawan et al. 1999 [16]	Thailand	37%
Elliott et al. 1993 [11]	Zambia	31%
Trajman et al. 1997 [19]	Brazil	30%
Aderaye et al. 1996 [5]	Ethiopia	22%
Frye et al. 1997 [2]	USA	11%
Gil et al. 1995 [12]	Spain	10%
Cordero et al. 1995 [7]	Spain	8%

**Table 3:** Reported yields of different tests in HIV-infected patients with pleural TB.

Study and year	Pleural fluid smear	*Pleural fluid culture	Pleural biopsy smear	*Pleural biopsy culture	Pleural biopsy histology	Sputum culture
Elliott al. 1993 [11]	0%	26%	NR	11%	52%	NR
Richter et al. 1994 [18]	6%	43%	20%	35%	86%	NR
Luzze et al. 2001 [15]	NR	43%	NR	NR	57%	NR
Conde et al. 2003 [26]	8%	15%	38%	77%	92%	77%
Hirsch et al. 2001 [27]	NR	87%	NR	NR	NR	NR
Kitinya et al. 1993 [35]	5%	5%	14%	14%	NR	NR
Heyderman et al. 1998 [13]	18%	NR	19%	42%	60%	NR

*Reported yields of cultures using Löwenstein-Jensen medium.

NR: not referred for test.
